# Recent advances in metathesis-derived polymers containing transition metals in the side chain

**DOI:** 10.3762/bjoc.11.296

**Published:** 2015-12-28

**Authors:** Ileana Dragutan, Valerian Dragutan, Bogdan C Simionescu, Albert Demonceau, Helmut Fischer

**Affiliations:** 1 Institute of Organic Chemistry, Romanian Academy, 202B Spl. Independentei, POBox 35-108, Bucharest 060023, Romania; 2 Petru Poni Institute of Macromolecular Chemistry, Romanian Academy, Iasi, Romania; 3 Macromolecular Chemistry and Organic Catalysis, Institute of Chemistry (B6a), University of Liège, Sart Tilman, Liège 4000, Belgium; 4 Department of Chemistry, University of Konstanz, Konstanz, Germany

**Keywords:** advanced materials, metallopolymers, metathesis, ROMP, transition metals

## Abstract

This account critically surveys the field of side-chain transition metal-containing polymers as prepared by controlled living ring-opening metathesis polymerization (ROMP) of the respective metal-incorporating monomers. Ferrocene- and other metallocene-modified polymers, macromolecules including metal-carbonyl complexes, polymers tethering early or late transition metal complexes, etc. are herein discussed. Recent advances in the design and syntheses reported mainly during the last three years are highlighted, with special emphasis on new trends for superior applications of these hybrid materials.

## Introduction

The fast growing interest in metal-containing polymers (metallopolymers) as advanced hybrid materials spurred prolific research in the worldwide organometallic and polymer scientific communities [1–4]. The variety of metals and the diversity of organic polymers allow tailoring metallopolymers so as to reach the desired physical and chemical properties suitable for progressive applications [5–7]. These functional hybrid materials are highly appreciated for their superior behaviour in catalysis, optics as well as for their magnetic, mechanical and thermal attributes. Structurally, metallopolymers are endowed with linear, cross-linked, hyperbranched, star or dendritic polymer architectures containing metals ranging from the main groups to transition metals and lanthanides which are embedded into the main chain or appended to the side chains of the polymer [8–11]. This make-up would confer an optimal set of capabilities that recommend them for diverse emerging application areas, e.g., as electro-optical and magnetic devices, for energy storage, nanomaterials, sensing, catalytic and drug-delivery systems [6,12–14].

Numerous synthetic routes have been explored to achieve the synthesis of these targets presently accessible through controlled and living polymerization techniques including controlled radical polymerizations (CRP) such as atom transfer radical polymerization (ATRP), nitroxide-mediated polymerization (NMP) and reversible addition–fragmentation chain transfer (RAFT) polymerization [15–16], living ionic polymerizations, specifically ring-opening polymerization (ROP) [17], as well as migration insertion polymerization (MIP) [18], acyclic diene metathesis polymerization (ADMET) [19–20] and ring-opening metathesis polymerization (ROMP) [21–27]. These synthetic strategies ensure metal incorporation from the corresponding metal-containing monomers into the polymer in a precise, predetermined mode. With the advent of new metathesis catalysts endowed with a high activity and chemoselectivity and good tolerance towards many functionalities [28–30], ROMP with Mo and Ru catalysts has become a very practical methodology in organic, polymer and materials chemistry. ROMP is also the method of choice for obtaining new and diverse metallopolymers [31–34].

The present contribution aims to provide an overview of selected developments in metathesis-based synthesis and applications of polymers containing transition metals in the side chain evidencing recent work published since our earlier review on this topic [34]. Metallopolymers are herein classified according to the nature of the transition metal and its binding mode to the organic moiety. Information on the physical characteristics of these materials is also included, with a focus on their present and future practical applications. Taking advantage of the considerable reactivity of ring-strained norbornenes and congeners and of their easy functionalization with many organic and organometallic groups it became possible to synthesize a broad range of polymers and copolymers by ROMP [35–36]. On the other hand it is well-known that ferrocene and numerous transition metal sandwich complexes exhibit great redox stability that allows fine tuning of their properties and applications in electrochemistry, sensing, catalysis, nanomaterials, etc. [37–40]. Not surprisingly, therefore, attention of researchers has turned first on metallopolymers containing ferrocene [33–34,41–43].

## Review

### Iron-containing polymers

Following the first successful application of Mo–alkylidene catalysts by Schrock and coworkers [42] in ROMP to ferrocene-appended monomers as well as the rapid expansion of Grubbs Ru metathesis catalysts [28–30], a vast number of iron-containing polymers have been synthesized by ROMP up to now [33–34,42–43].

**Scheme 1:**
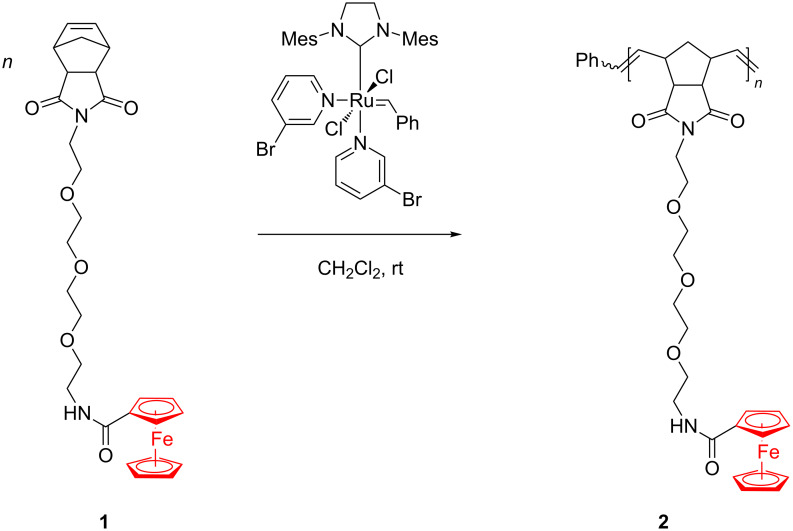
Synthesis of homopolymers containing ferrocenyl and tetraethylene glycol groups.

In a compelling work, Astruc et al. [44] reported a biologically relevant type of new homopolymers (e.g., **2**, Scheme 1) and block copolymers provided with amidoferrocenyl groups linked through a tetraethylene glycol side chain. These interesting metallopolymers were readily prepared through living ROMP initiated by the Grubbs 3rd generation catalyst which proved quite active and tolerant toward the monomer endowed with multiple functionalities (Scheme 1).

By precisely controlling the living polymerization process, they succeeded in varying the number of amidoferrocenyl motifs in the polymers within pre-established limits. Such polymers and block copolymers were used to prepare modified Pt electrodes with high stability and good qualitative sensing of ATP^2−^ anions. It was supposed that the triethylene glycol domains in the block copolymers favor the amidoferrocene–ATP interactions by encapsulation. Astruc assumed that during the recognition process different H-bonding modes arise in the supramolecular polymeric network, i.e., an intramolecular H bonding with the β- and γ-phosphate groups of ATP^2−^ and an intermolecular H bond between the α-phosphate and another amidoferrocenyl group. Redox properties of polycationic copolymers containing the complex [Fe(η^5^-C_5_H_5_)(η^6^-C_6_Me_6_)][PF_6_] have been recently revealed as potential electron-transfer reagents provided with a high stability [45].

**Scheme 2:**
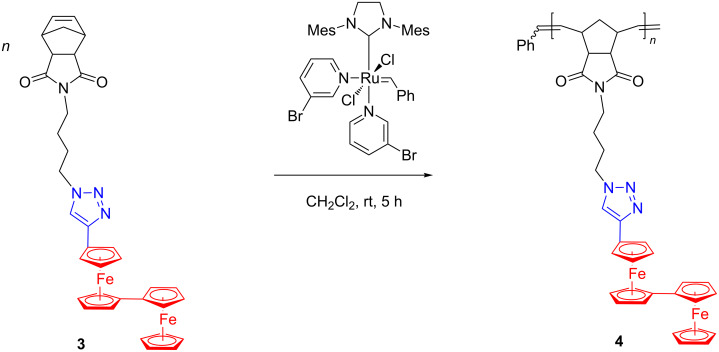
Synthesis of redox-robust triazolylbiferrocenyl polymers **4**.

On extending their research to the areas of anion sensing and nanomaterials, the Astruc group accomplished an efficient synthesis, by ROMP with Grubbs 3rd generation catalyst, of redox-robust triazolylbiferrocenyl (trzBiFc) polymers **4** bearing the organometallic group in the side chain (Scheme 2) [46–47].

Here again, the Grubbs 3rd generation catalyst was very active and highly tolerant towards the biferrocene and triaza functionalities. Noteworthy, the oxidation of the polymer **4** with ferricenium hexafluorophosphate led to a stable biferrocenium polymer while oxidation with Au(III) or Ag(I) allowed the formation of networks with nanosnake morphology, consisting of mixed-valent Fe(II)–Fe(III) polymers that encapsulate metal (Au or Ag) nanoparticles (NPs). These polymers were suitable for obtaining modified Pt electrodes with good sensing affinities for ATP^2−^ and Pd(II) cations. The importance of such results lies in the multi-electron properties of these side-chain BiFc polymers that have not been much studied so far although the outstanding stability of the biferrocenium motifs recommends them for designing new redox reactions, eventually leading to value-added nanomaterials. Along a different line, in a recent, inventive work Astruc and coworkers [48] demonstrated that triazolylbiferrocenyl-containing polymers can effectively stabilize palladium nanoparticles (PdNPs) affording highly active catalysts for Suzuki–Miyaura coupling reactions.

### Cobalt-containing polymers

**Scheme 3:**
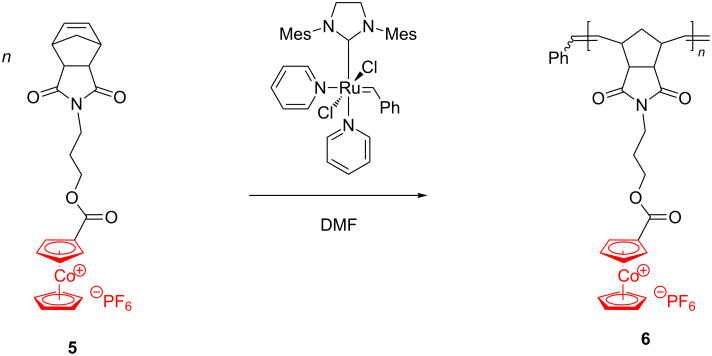
Synthesis of cobaltocenium-containing polymers by ROMP.

The incorporation of other late transition metals such as cobalt into polymers soon emerged as an efficient and rapid method for the production of nanostructured materials of scientific and practical importance for microelectronics, catalysis, biology and medicine (vide infra). Tang et al. [49] were the first to apply the ROMP strategy to synthesize the well-defined, high molecular weight cobaltocenium-containing polymer **6** (Scheme 3).

**Scheme 4:**
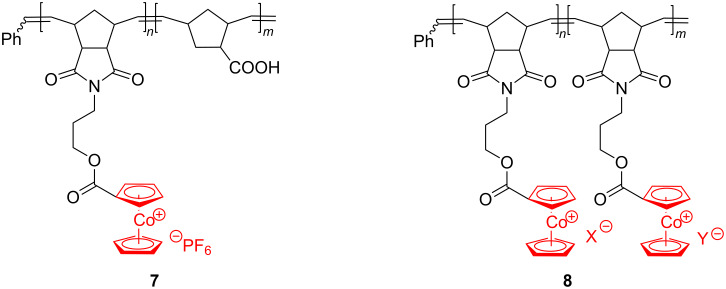
Cobaltocenium-appending copolymers by the ROMP approach (X = PF_6_, Y = BPh_4_ or Cl).

Under ambient conditions, the Grubbs 3rd generation catalyst induced polymerization of **5** in a living manner leading to a product with low polydispersity (1.12) and high molecular weight (167,000 g·mol^−1^). By substituting the PF_6_^−^ anion with BPh_4_^−^, Cl^−^ or an anion exchange resin (chloride-form), the authors demonstrated that the nature of the anion is important for the polymer properties. They found that polymer **6** was soluble in water and various organic solvents when the counteranion was chloride. Subsequently, these authors copolymerized **6** with norbornene-2-carboxylic acid, using Grubbs 3rd generation catalyst, to prepare diblock copolymer **7**, in which one block contains cobaltocenium units while the other block comprises an organic chain only [50] (Scheme 4).

By the same technique, polymer **6** was further copolymerized with a cobaltocenium-BPh_4_ monomer and a cobaltocenium-Cl monomer affording, respectively, the new diblock copolymers **8** (X **=** PF_6_, Y = BPh_4_ or Cl). Self-assembly of these block copolymers into core–shell spherical micelles was successfully conducted and, by UV/ozonolysis or thermal pyrolysis generating antiferromagnetic CoO species, some of these micelles could be converted into inorganic nanoparticles.

**Scheme 5:**
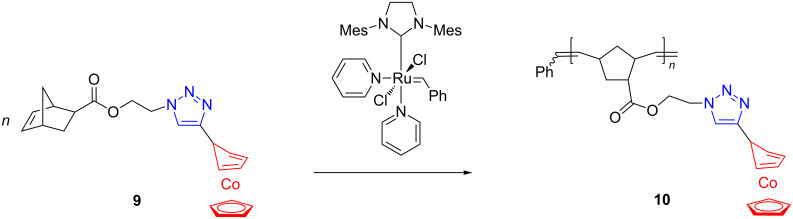
Cobalt-containing polymers by click and ROMP approach.

With the aim at extending the application of metallopolymers as heterogeneous macromolecular catalysts for living radical polymerizations, Tang et al. [51] produced the cobalt-containing polymer **10** by ROMP of the norbornene monomer **9**, derivatized with triazolyl and cyclopentadienylcobalt-1,3-cyclopentadiene moieties (Scheme 5).

The triazolyl unit was first attached to the η^4^-cyclopentadiene CpCo(I) complex by click reaction of the corresponding alkyne precursor and then the triazolyl-Co scaffold was incorporated into the norbornene monomer **9** by conventional esterification. It is important to note that the cyclopentadienyl-cobalt-1,3-cyclopentadiene, an isoelectronic 18-electron species to ferrocene and cobaltocenium, was well tolerated by the Grubbs 3rd generation ROMP catalyst. The polymerization of **9** proceeded in a controlled and living manner under ambient conditions. Polymer **10** was successfully employed as an organometallic catalyst in the atom-transfer radical polymerization of methyl methacrylate or styrene to obtain poly(methyl methacrylate) and polystyrene devoid of colored traces of catalyst, a very important requirement for special applications, e.g., in dentistry, medical devices, housewares, and food packaging. In another recent study, Tang and coworkers [52] performed a quantitative analysis of counterion exchange in cobaltocene-containing polyelectrolytes that are accessible by an initial ROMP, and subsequently derivatized with cobalt motifs. These results appear to be relevant for self-assembly and drug-delivery systems with this type of polyelectrolytes.

**Scheme 6:**
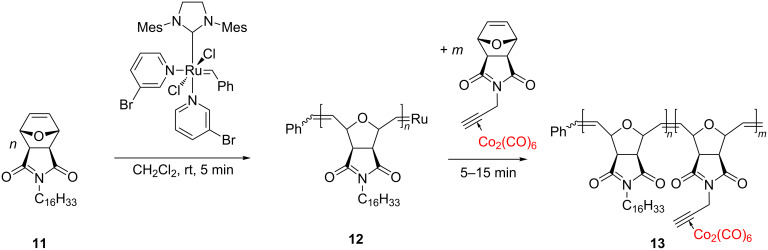
Synthesis of new cobalt-integrating block copolymers.

An interesting cobalt-containing diblock copolymer, bearing a dicobalt hexacarbonyl complex coordinated to an alkyne, with a constant block ratio was proposed as a new magnetic material by Tew et al. [53]. Their procedure involved the synthesis of a first block polymer, **12**, by ROMP of monomer **11** using Grubbs 3rd generation catalyst. The second block polymer was created by the addition of the cobalt-containing monomer to the reaction mixture containing **12** to continue the ROMP. Diblock copolymer **13**, with a defined block ratio, could be obtained by the variation of the polymerization time (Scheme 6).

In this process, the ruthenium initiator proved to well tolerate the dicobalt hexacarbonyl complex embedded in the monomer. By controlled heating of the cobalt-containing block copolymers, robust, room temperature ferromagnetic (RTF) materials have been obtained.

**Scheme 7:**
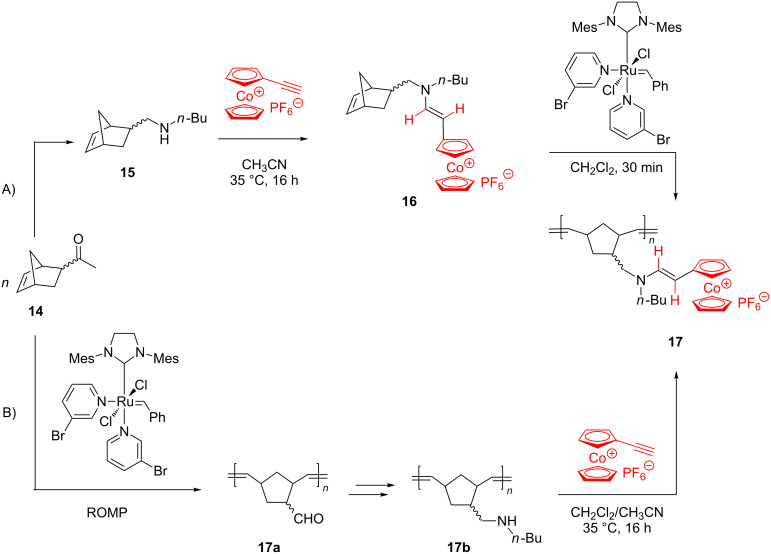
Two alternative routes for the synthesis of redox-active cobalticenium-tethered polyelectrolytes.

By two alternative ROMP protocols, both starting from 5-formyl-2-norbornene (**14**) and using the Grubbs 3rd generation catalyst, Astruc and coworkers [54] successfully prepared new redox-active cobalticenium-tethered polyelectrolytes of type **17**. According to the first protocol, the norbornene monomer containing an enamine-cobalticenium group (**16**) was first prepared by hydroamination of the ethynyl cobalticenium with *n*-butylamine-substituted norbornene **15**. Next, **16** was polymerized to **17**, by ROMP under mild conditions (Scheme 7A). In the second approach, first, the monomer **14** was polymerized to **17a**, followed by functionalization of the latter with *n*-butylamine to yield **17b**, and finally this organic polymer hydroaminated the ethynyl cobalticenium to produce **17** (Scheme 7B). Both protocols embody an elegant and original ROMP-based access to cobalticenium-containing polyelectrolytes.

### Ruthenium-, iridium-, osmium- and rhodium-containing polymers

**Scheme 8:**
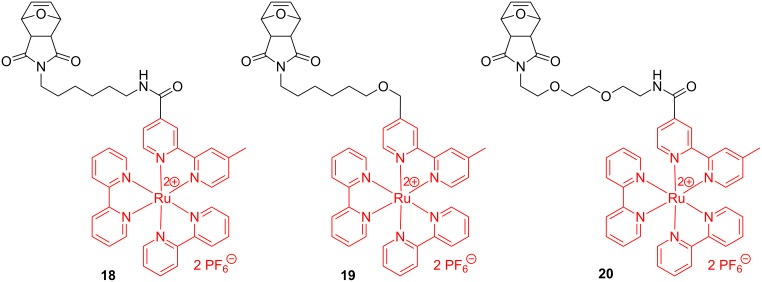
Oxanorbornene monomers for the synthesis of Ru-containing polymers by ROMP.

**Scheme 9:**
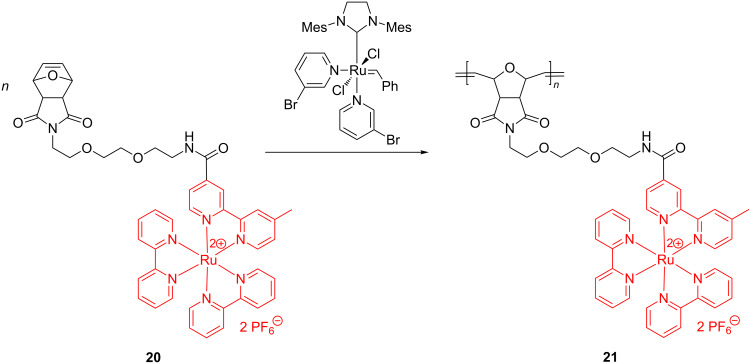
ROMP synthesis of Ru-containing homopolymers.

**Scheme 10:**
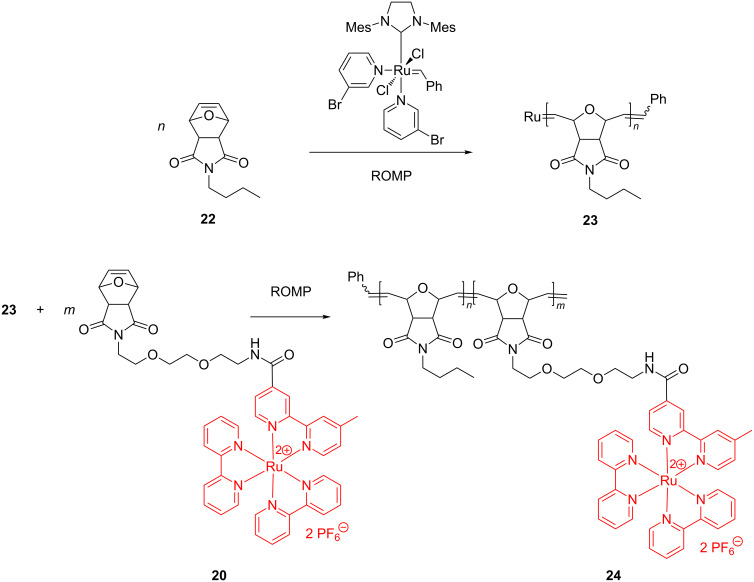
Synthesis of diblock copolymers incorporating ruthenium.

**Scheme 11:**
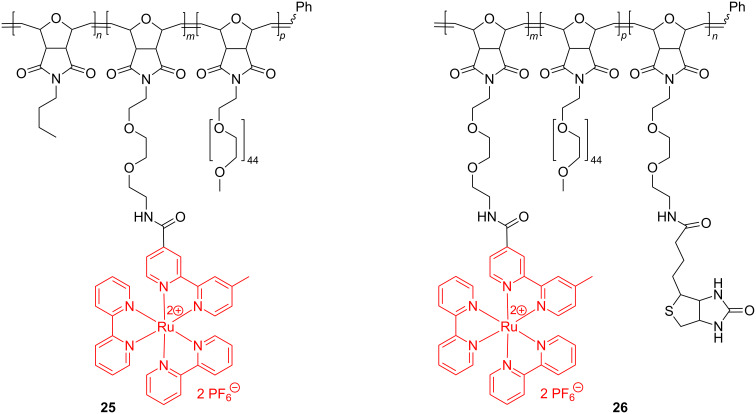
Synthesis of Ru triblock copolymers.

ROMP syntheses of homopolymers and block copolymers bearing bipyridine–ruthenium complexes starting from norbornene or oxanorbornene functionalized with Ru complexes have been reported by several authors [55–56]. In these investigations it was revealed that the Ru catalysts are active initiators in producing, in a living polymerization manner, well-defined polymers containing Ru in the side chains. Again, the best results were obtained with the Grubbs 3rd generation catalyst. Along this line, Sleiman et al. [56] prepared an array of oxanorbornene monomers tethered with ruthenium–bipyridine motifs (e.g., **18**–**20**, Scheme 8) and used them to prepare homopolymers (Scheme 9), diblock- (Scheme 10) and triblock copolymers (Scheme 11).

In an in-depth exploration of the synthesis of diblock copolymers **24**, Sleiman developed a step-wise procedure: in the first step, the ruthenium catalyst induced polymerization of the bicyclic monomer **22** to homopolymer **23**, followed by polymerization of the additional comonomer **20** at the Ru site of **23** to yield the copolymer **24** (Scheme 10).

Based on their potential application as tools for biological detection and signal amplification, amphiphilic Ru-modified triblock copolymers have been produced from biocompatible and bioconjugatable oxanorbornene monomers. By extending the above ROMP methodology, Sleiman et al. managed to synthesize the Ru triblock copolymers **25** and **26** (Scheme 11), and examined their self-assembling into micelles in aqueous media to evaluate them as luminescent markers of biological molecules.

**Scheme 12:**
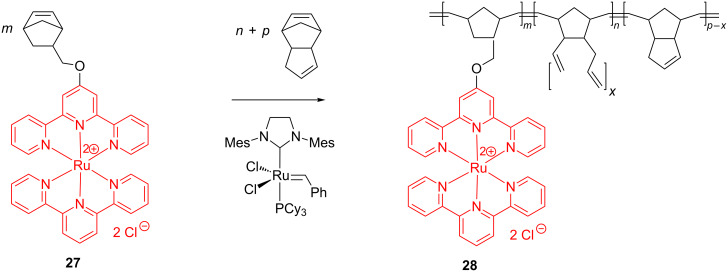
Synthesis of cross-linked Ru-containing triblock copolymers.

The production of metal-cation-based anion exchange membranes from ROMP polymers was first reported by Tew et al. [57]. The ROMP reaction, induced here by the Grubbs 2nd generation catalyst, implied the copolymerization of a norbornene monomer (**27**) functionalized with a water-soluble bis(terpyridine)ruthenium(II) complex, with dicyclopentadiene as a cross-linking agent (Scheme 12). In the resulted copolymer **28** each Ru complex is associated with two counteranions (chloride), which represents a novelty versus most cation-based membranes provided with single cation–anion pairs. Cross-linking with dicyclopentadiene ensured a high mechanical stability of the copolymer. The film cast from **28** displayed an anion conductivity and mechanical properties similar to those of the traditional quaternary ammonium-based anion exchange membranes. In addition, the film exhibited high methanol and base tolerance making it suitable for applications in fuel cells and anion-conducting devices.

**Scheme 13:**
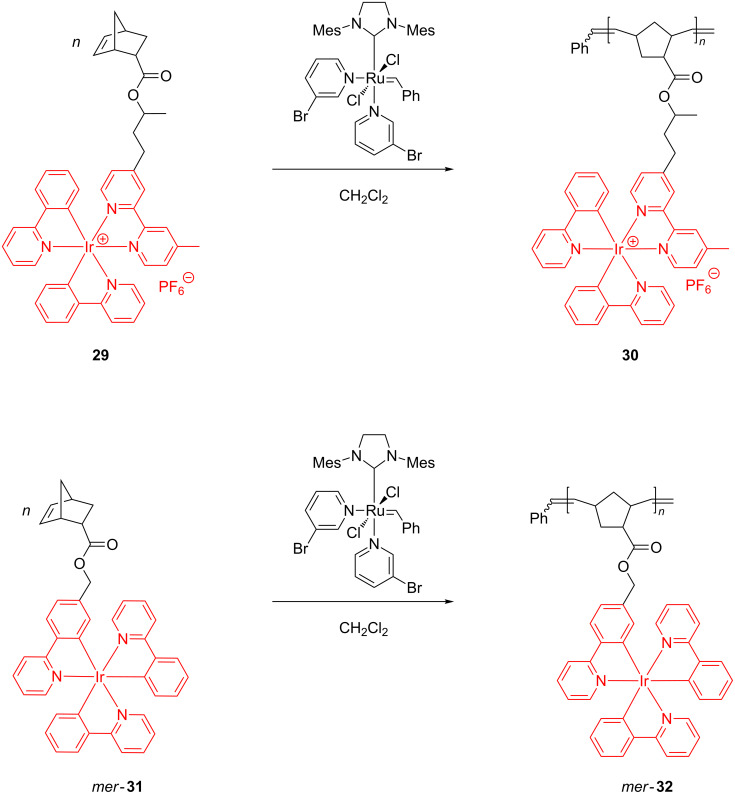
Synthesis of Ir-containing homopolymers by ROMP.

Owing to their high phosphorescent propensity, complexes based on iridium have been grafted onto polymers for the application as light-emitting diodes (LEDs) [58]. In an earlier research, in order to obtain iridium-containing polymers by the ROMP route, Weck and coworkers [59] polymerized monomers **29** and *mer*-**31**, in the presence of Grubbs 3rd generation catalyst, to the fully soluble ROMP homopolymers **30** and *mer*-**32** (Scheme 13).

**Scheme 14:**
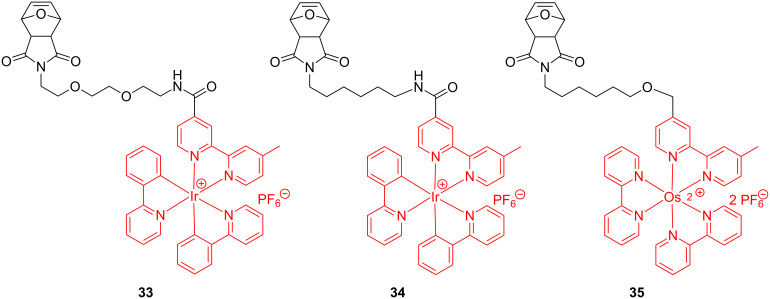
Monomers for Ir- and Os-containing ROMP polymers.

Later on, while investigating the self-assembly of transition metal-containing polymers, Sleiman et al. [60] expanded the field by preparing ROMP-able oxanorbornene monomers having iridium and osmium bipyridines attached by an extended organic linker (Scheme 14).

**Scheme 15:**
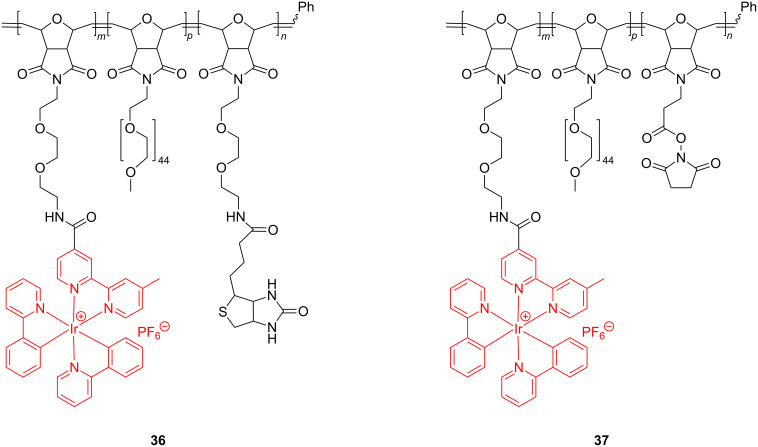
ROMP block copolymers integrating Ir in their side chains.

The triblock copolymers obtained through a sequential ROMP of the corresponding monomers, appended to Ir bipyridine complexes, oligoethylene glycol and biotin entities, have been examined by fluorescence spectroscopy for their self-assembling behavior and biodetection capability (Scheme 15).

**Scheme 16:**
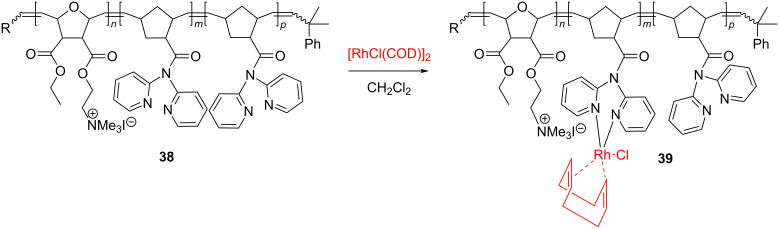
Synthesis of Rh-containing block copolymers.

In a very interesting work, Blechert, Buchmeiser and coworkers [61] copolymerized norborn-5-ene-(*N*,*N*-dipyrid-2-yl)carbamide with *exo*,*exo*-[2-(3-ethoxycarbonyl-7-oxabicyclo[2.2.1]hept-5-en-2-carbonyloxy)ethyl]trimethylammonium iodide to polymer **38**, using the Schrock Mo catalyst. By further reaction with [Rh(COD)Cl]_2_ (COD = cycloocta-1,5-diene), polymer **38** gave the Rh(I)-appended block copolymer **39** (Scheme 16).

Remarkably, in water, this Rh-containing block copolymer readily generated micelles and could be thus successfully employed as a Rh-immobilized catalyst for the hydroformylation of 1-octene.

**Scheme 17:**
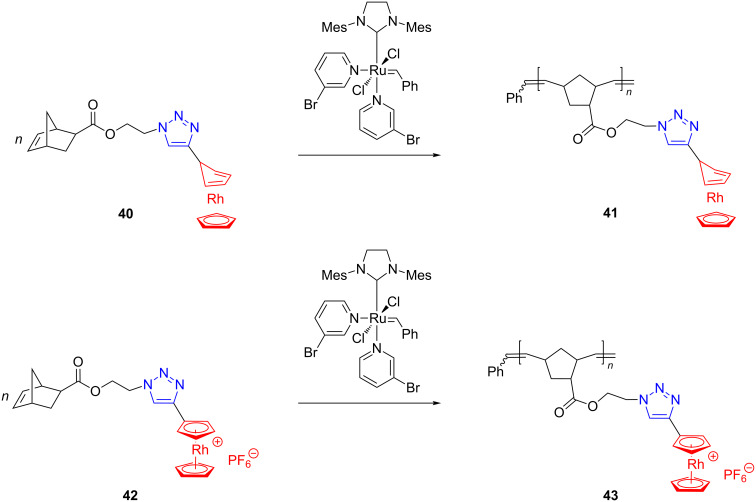
Access to rhodocenium-containing metallopolymers by ROMP.

Very recently, Matyjaszewski, Tang and coworkers [62] reported the first synthesis of norbornene monomers substituted with rhodocenium units and their controlled polymerization, by two parallel routes (ROMP and RAFT), to rhodocenium-containing metallopolymers. ROMP of both triazolyl-rhodocenium monomers, **40** and **42**, proceeded productively and in a living fashion to yield amphiphilic metallopolymers **41** and **43** (Scheme 17).

Polymers **41** and **43** have been evaluated for their counterion exchange properties and self-assembling tendency revealing a promising application profile. The point of interest here is that rhodocenium exhibits different chemical and physical properties from cobaltocenium. A novel immobilized Rh catalytic system in which the metal is embedded, by means of the 5,5-dinorimido BINAP ligand, into the polymer, obtained from alternating ROMP of cyclooctene with the Grubbs first generation catalyst, has been disclosed in a patent by Bergens et al. [63]. This catalytic system allowed the intramolecular cycloisomerization of enynes with high yields and turnover numbers.

### Copper-containing polymers

**Scheme 18:**
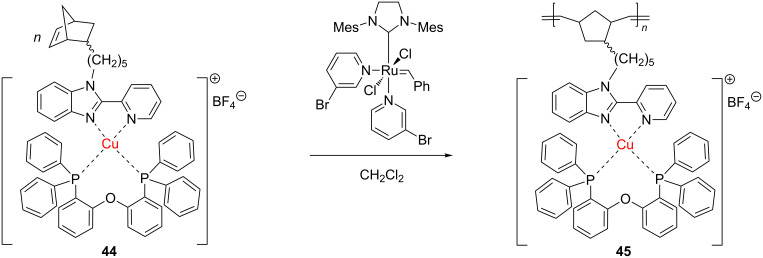
Synthesis of homopolymers equipped with Cu coordination centers.

**Scheme 19:**
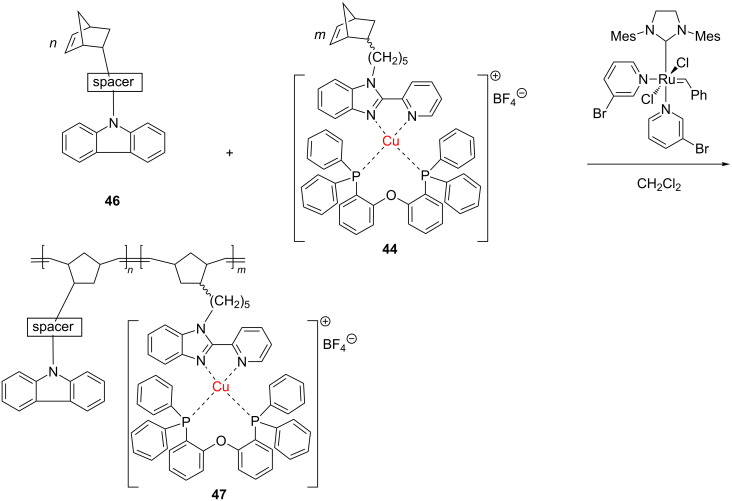
Synthesis of Cu-containing copolymers (spacer = –(CH_2_)_5_–; >C=O).

A copper(I) complex containing a norbornene substituted with the 2-(pyridin-2-yl)-1*H*-benzimidazole ligand, **44**, developed by Il'icheva et al. [64], came to the attention of the scientific community involved in the area. The complex was used to access Cu-containing homopolymers **45** and copolymers **47** under metathesis polymerization with the Grubbs 3rd generation catalyst (Scheme 18 and Scheme 19). Further variations in the spacer subunit from a norbornene carbazole comonomer **46** enabled fine-tuning of the physical and chemical properties of the copolymer **47**.

These materials, in which Cu is tethered to the polymeric backbone by an organic linker, exhibited notable luminescent characteristics. The same research group subsequently introduced other new copper(I) complexes, ligating norbornene-substituted phenanthroline, that were polymerized by ROMP (Grubbs 3rd generation catalyst) to yield copolymers with valuable photo- and electroluminescent properties [65]. This kind of hybrid structure may induce high performance in LED devices.

### Early transition metal-containing polymers

**Scheme 20:**
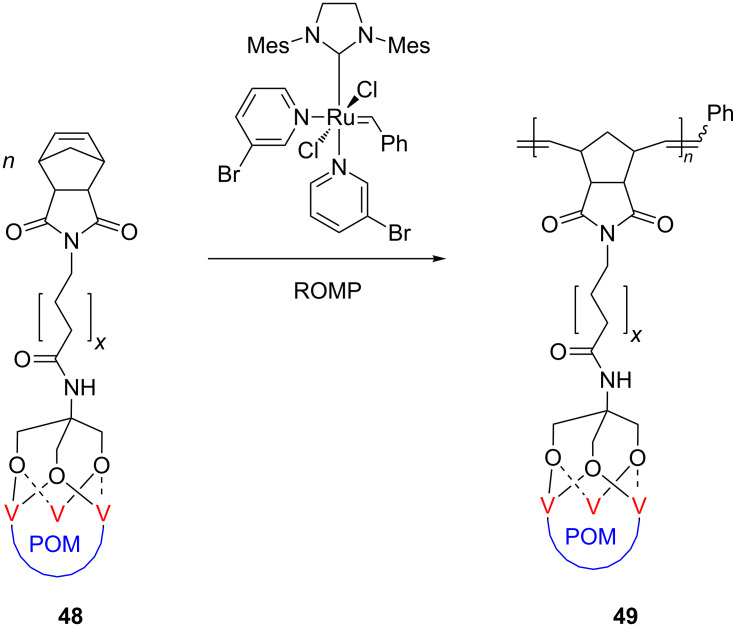
Synthesis of polynorbornene bearing a polyoxometalate (POM) cluster in the side chain.

In contrast to the numerous polymers including late transition metals discussed so far, only few representatives of early transition metals attached to ROMP polymers have been disclosed recently. Thus, Wang et al. [66] communicated the ROMP synthesis of the first polynorbornene bearing a polyoxometalate (POM) cluster in the side chain (Scheme 20).

According to their concept, the norbornene monomer containing a trivanadium-substituted Wells–Dawson-type polyoxotungstate (POM) (**48**) was polymerized quantitatively to **49** in a living and controlled process under promotion of Grubbs 3rd generation catalyst. It should be remarked that this Grubbs catalyst favored the polymerization under mild reaction conditions and tolerated very well the bulky POM cluster attached to the monomer. The obtained hybrid materials are promising candidates for the production of high-performance catalysts based on poly(polyoxometalate)s.

### Lanthanide-containing polymers

**Scheme 21:**
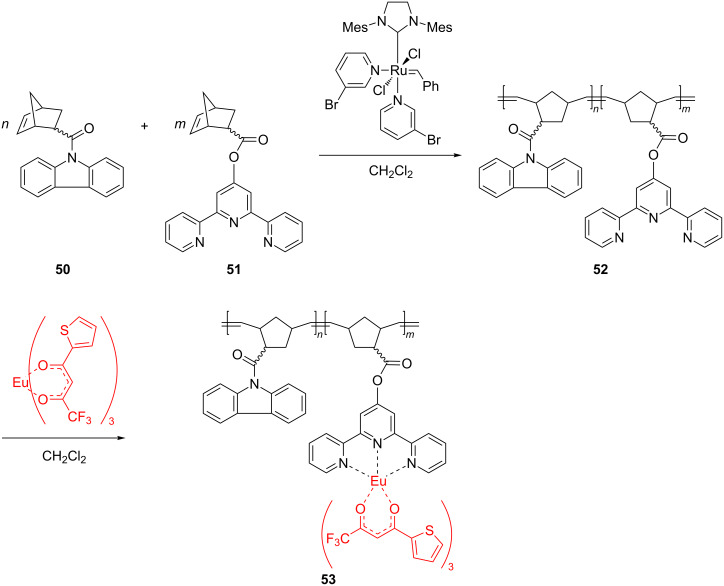
Synthesis of Eu-containing copolymers by a ROMP-based route.

Recently, new polynorbornenes of type **53**, functionalized with terpyridine and carbazole moieties and integrating a europium complex in the pendant chains, were described by Rozhkov et al. [67]. They were obtained by a metathesis copolymerization with Grubbs 3rd generation catalyst as the key step (Scheme 21).

In a first approach the copolymer **52** was coordinated with europium thenoyl trifluoroacetonate to yield copolymers **53** with different ratios between the purely organic and europium-containing units. Alternatively, similar coordination copolymers were prepared by copolymerizing the europium complex of the terpyridine monomer **51** with the carbazole-substituted norbornene **50**. In solution or in thin film these Eu-containing products exhibited important metal-centered photoluminescence recommending them for novel applications.

Unveiling and rationalizing the interactions between the metal and the organic polymer backbone and/or side chains is crucial for ensuring the desired properties for the hybrid material [68]. Indeed, when appraising luminescence of a series of polynorbornenes attaching various homoleptic bi- or trinuclear lanthanide salen complexes (with La, Nd, Yb, Er, Gd or Tb), Lü et al. [69–70] established that, only in the case of Nd and Yb metallopolymers, the luminescent emissions are strongly retained versus those of the respective monomers in solution.

## Conclusion

This review highlights ingenious ways in which a large variety of transition metals could be attached to organic polymer side-chains thus prompting the appearance of extraordinary new physical properties (optical, electrical, conducting, catalytic, magnetic, biological, etc.), most of which were not detained before by either the metal or the organic counterpart. Such distinguishing features recommend these privileged scaffolds as important hybrid materials having a strong impact on a host of current high-tech applications, as fuel cells, light-emitting diodes (LED), magnetic nanomaterials, catalysts, biosensors, for energy generation and storage. The mainstay of the synthesis of these engineered metallopolymers is living ROMP, the key step advantageously executed either with Schrock’s or Grubbs latest generation catalysts, and easier to be precisely controlled versus other techniques used for the preparation of metallopolymers.
